# Evidence based processes to prevent readmissions: more is better, a ten-site observational study

**DOI:** 10.1186/s12913-021-06193-x

**Published:** 2021-03-01

**Authors:** Jacqueline Pugh, Lauren S. Penney, Polly H. Noël, Sean Neller, Michael Mader, Erin P. Finley, Holly J. Lanham, Luci Leykum

**Affiliations:** 1 Department of Internal Medicine, University of Texas Health at San Antonio, Long School of Medicine, San Antonio, TX USA; 2 South Texas Veterans Health Care System, Research Service, San Antonio, TX USA; 3 Department of Family and Community Medicine, University of Texas Health at San Antonio, Long School of Medicine, San Antonio, TX USA; 4 Department of Internal Medicine, University of Texas at Austin, Dell Medical School, Austin, TX USA

**Keywords:** Hospital readmissions, Transitions of care processes, Quality of care

## Abstract

**Background:**

30-day hospital readmissions are an indicator of quality of care; hospitals are financially penalized by Medicare for high rates. Numerous care transition processes reduce readmissions in clinical trials. The objective of this study was to examine the relationship between the number of evidence-based transitional care processes used and the risk standardized readmission rate (RSRR).

**Methods:**

Design**:** Mixed method, multi-stepped observational study. Data collection occurred 2014–2018 with data analyses completed in 2021. Setting: Ten VA hospitals, chosen for 5-year trend of improving or worsening RSRR prior to study start plus documented efforts to reduce readmissions. Participants**:** During five-day site visits, three observers conducted semi-structured interviews (*n* = 314) with staff responsible for care transition processes and observations of care transitions work (*n* = 105) in inpatient medicine, geriatrics, and primary care. Exposure**:** Frequency of use of twenty recommended care transition processes, scored 0–3. Sites’ individual process scores and cumulative total scores were tested for correlation with RSRR. Outcome**:** best fit predicted RSRR for quarter of site visit based on the 21 months surrounding the site visits.

**Results:**

Total scores: Mean 38.3 (range 24–47). No site performed all 20 processes. Two processes (pre-discharge patient education, medication reconciliation prior to discharge) were performed at all facilities. Five processes were performed at most facilities but inconsistently and the other 13 processes were more varied across facilities. Total care transition process score was correlated with RSRR (R^2^ = 0..61, *p* < 0.007).

**Conclusions:**

Sites making use of more recommended care transition processes had lower RSRR. Given the variability in implementation and barriers noted by clinicians to consistently perform processes, further reduction of readmissions will likely require new strategies to facilitate implementation of these evidence-based processes, should include consideration of how to better incorporate activities into workflow, and may benefit from more consistent use of some of the more underutilized processes including patient inclusion in discharge planning and increased utilization of community supports. Although all facilities had inpatient social workers and/or dedicated case managers working on transitions, many had none or limited true bridging personnel (following the patient from inpatient to home and even providing home visits). More investment in these roles may also be needed.

## Background

Early hospital readmissions (≤30 days following discharge) were initially proposed as an indicator of quality of care in the 1980s and have since been used as a quality metric by Medicare and the Department of Veterans Affairs [1–4]. Besides its use as a measure of quality, readmissions can have financial consequences for hospitals. The Affordable Care Act included the Hospital Readmission Reduction Program (HRRP) through which hospitals with excess readmissions receive reduced Medicare payments [5]. Additionally, accountable care organizations and other financially at-risk delivery systems view readmission reduction as a strategy to reduce costs [6].

The degree to which early readmissions are preventable has been debated, with estimates of the proportion of readmissions that are preventable ranging from 15 to 45% [7, 8], but there is growing consensus regarding the need for improved transition of care interventions to reduce preventable readmissions [9, 10]. Coordination of care transitions between inpatient, outpatient and home for patients can be complex and expensive [10]. Numerous trials of single, combined, and complex interventions to facilitate care transitions and thereby reduce admissions have been conducted [11–13]. Systematic reviews of this literature have summarized the most effective processes such as patient education, medication reconciliation, discharge planning, and post-discharge phone calls [14–17]. Leppin et al. [14] found in subgroup analyses that multicomponent interventions, spanning multiple care staff, that included supports for patient self-care were more effective at reducing readmissions. Burke et al. [18], using the domains of the Ideal Transitions In Care Framework, found that increasing the number of domains included in a given intervention significantly increased success at reducing readmissions. Similarly, Bradley et al. [19]_,_ in CHF care transitions, found that hospitals that implemented more strategies had lower readmission rates. Risk-adjusted readmission rates have fallen significantly since the late 1990s in the VA [20]and in Medicare hospitals since 2012 [21], likely reflecting investment in improved care transition processes and post-discharge care as well as improved coding of comorbidities for risk adjustment and increased use of observation status [22, 23].

As health care systems, hospitals and accountable care organizations have moved towards implementation of transitional care processes to reduce readmissions, less is known about what specific processes are being implemented or what combinations of processes are most effective in practice. This mixed method case study of 10 Department of Veterans Affairs (VA) hospitals sought to describe the range of transitional care processes offered and their effect on risk adjusted readmission rates.

## Methods

### Design

This mixed method, multi-stepped observational study used concurrent triangulation of data from 10 VA hospitals across the US. Sites were chosen based on five years of either improving or worsening readmission rates prior to study start and documented efforts to reduce readmissions. The full protocol, including qualitative interview guides developed for this study, data collection and analysis, is described in depth elsewhere [24]. Four trained, experienced investigators (one physician with 30 years experience in health services research and qualitative studies [JP], one PhD medical anthropologist with 10 years experience [LP], one PhD behavioral psychologist with 25 years experience in health services research, and one PhD [HL] in business with 15 years experience in qualitative studies) collected the data. Other than employment by the VA, the investigators held no known biases regarding readmissions. Investigators had no previous relationships with participants prior to study start. All investigators were oriented and trained on data collection materials and participated in pilot testing and refining of the data collection tools at our home research facility.

During five-day site visits, three observers conducted semi-structured interviews and observations of care transitions work in inpatient medicine, geriatrics, specialty clinics providing care management or hospital follow-up care, and primary care. All interviews were audio recorded and field notes were taken of the observations. Interviews were typically 20–60 min long. Focus groups were typically 45–60 min. Data were obtained from various staff responsible for care transition processes, including nurses, pharmacists, doctors, and discharge-coordinating staff. We purposively sampled across the facilities to ensure heterogeneity in position (e.g., frontline staff, facility leadership), service (e.g., inpatient, primary care, geriatrics), and role (e.g., nursing, physicians, quality improvement). Individuals were identified to participate in the study through key personnel we identified based on organizational charts (e.g. service leaders) and snowball sampling. Participants in interviews and focus groups were invited to participate through email or face-to-face. Interviews were conducted with 21–41 staff members at each site (*n* = 314). We also conducted semi-structured observations of 45 interdisciplinary team or discharge planning meetings with over 1200 patient discussions, 37 individual patient discharge instructions (26 unit RN, 9 PharmD, 3 Social Worker, and 1 RD (some patients were instructed by more than one role)), and more than 60 physician team rounds, with observations documented in templated and/or field notes (described in protocol paper [24]), depending on the setting. Refusal to participate was rare with reasons most often due to time limitations during the study visit dates.

At the conclusion of each site visit, a care transitions process checklist derived from the readmissions literature and created prior to any site visits was used to assess processes at each facility. This list consisted of 20 processes which have been shown to reduce readmissions alone or in combination and which addressed both medical as well as psychosocial factors [14–17]. Investigators who participated in the site visits met and identified all the established care transition processes they observed or that were described to them, and subsequently performed member checking at outbriefing meetings with hospital leadership. Each site’s checklist was further confirmed after analysis of qualitative interview and observation data, referring to transcripts, audio recordings, meeting notes and observational results as necessary. Consensus was achieved among the three reviewers for all sites. NVIVO was used for qualitative data coding and the process for the larger study is described in depth in the protocol paper [24].

Table 1 lists the 20 processes, the operational definitions and checklist scoring for each process. Investigators scored all checklist processes except the post-discharge call on a 0 to 3 scale: 0, facility does not perform this process; 1, this process is performed inconsistently; 2, this process is performed consistently but only for certain disease specific or high-risk patient groups; 3, this process is done for all patients who meet criteria for receiving this process but is not disease dependent. See Table 1 for details on scoring for individual processes. Post discharge call completion rate was scored using performance data from the VA Primary Care Almanac [25].

**Table 1:** Transitional Care Processes Assessed

Process	Definition	Scoring
Pre-discharge patient education	Patient given instructions by staff (nurses, physicians, pharmacists, social workers, etc.) about the reasons for their admission, their disease state, their medications, what they need to do at home, warning signs or red flags, who to call, follow-up, etc.	0 = facility does not perform this process 1 = this process is performed inconsistently 2 = this process is performed consistently but only for certain disease specific patient groups 3 = this process is done for all patients who meet criteria for receiving this process but is not disease dependent
Use of teach-back method with patients	The teach-back method involves having the patient or caregiver who received the teaching, explain back to the teacher what they understood in order to check comprehension of information learned.	0 = did not observe or mention in discussions 1 = mentioned in discussions or on template but not observed 2 = mentioned and observed but not consistently 3 = many patient-staff observations included teach-back
Increased emphasis on patient education about diagnoses, self-management and medications throughout hospitalization	Education delivered during hospital stay by video, group classes or one-on-one to patients in addition to standard discharge instructions.	0 = did not observe or mention in discussions 1 = mentioned in discussions but not observed 2 = mentioned and observed 3 = programs individualized to patient needs and observed consistently across multiple different staff roles
Communication of medical plans in front of patients during physician team rounds	Discussions held in patient rooms and preferably engaging patients themselves regarding diagnostic and treatment decision making and plans by inpatient staff including physician teams, nurses, and other team members. Note: In this study only physician team rounds were observed systematically.	0 = rounding in the team room 1 = bedside rounds but discussions occurring in hall way or team room 2 = almost all discussions in patient room but with physicians only 3 = multidisciplinary team discussions in patient room
Implementation of a discharge checklist	Checklist of items to be considered prior to discharging a patient such as living situation, need for prosthetic items, need for home health, availability of a caregiver, transportation needs to come back to appointments.	0 = facility does not have a checklist 1 = existence of checklist mentioned but not used consistently 2 = this process is performed consistently but only for certain patient groups such as palliative care 3 = checklist used for all patients being discharged
Assessment of readmission risk	Either calculating readmission risk with a risk calculator or assessing a list of risk factors prior to discharge if they exist (such as Project BOOST 8P)	0 = facility does not perform this process 1 = this process is performed inconsistently 2 = this process is performed consistently but only for certain disease specific patient groups 3 = this process is done for all patients and is not disease dependent.
Implementation of discharge planning rounds	Multidisciplinary meetings with physicians, nurses, case managers, social workers,	0 = facility does not perform this process 1 = inconsistently done (either frequency or coverage of only a portion of patients) 2 = done for most pts. with an emphasis on high risk 3 = done for all patients 5 days a week with a multidisciplinary team
Medication reconciliation prior to discharge	Medications are reviewed prior to discharge to insure that all medication changes (new drugs, dose change on previously prescribed drugs and elimination of drugs) are accurate in medical record..	0 = facility does not perform this process 1 = this process is performed inconsistently 2 = this process is performed consistently but only for certain disease specific patient groups 3 = this process is done for all patients who meet criteria for receiving this process but is not disease dependent.
Assignment of medication reconciliation to pharmacist	Pharmacist rather than physician or other staff member performs the above reconciliation	0 = facility does not use pharmacists to complete med rec 1 = this process is performed inconsistently by pharmacists 2 = this process is performed consistently by pharmacists but only for certain disease specific patient groups 3 = this process is done for all patients by pharmacists
Utilization of discharge/care transitions case manager	Logistical inpatient care coordination to insure that pt. leaves with equipment needed in the home, that appointments are made, that home health is ordered, that transportation to home is available. If pt. to go to SNF or NH, then additional work on funding, acceptance, and orders must be done.	0 = facility does not have discharge or care transitions case managers 1 = has case managers but not available consistently or for all patients 2 = case manager for all patients 3 = has case managers for all patients plus transitional care managers for high risk groups
Printed follow-up instructions which might include medication reconciliation, follow-up appointments, self-care tasks or action plan for management of symptoms	Pt given a printed set of discharge instructions that includes the new medication list, their follow-up appointments, self-care tasks and action plan for symptom deterioration. Presented by nurse and/or pharmacist with verbal instructions prior to leaving hospital.	0 = facility does not perform this process 1 = this process is performed inconsistently 2 = has standardized discharge package for all patients 3 = has additional patient-centered discharge instructions for patients
Post discharge follow-up appointments to PCP and for diagnostic testing made prior to discharge	Appointments made and given to patient prior to patient leaving to go home. These could be for primary or specialty care or for diagnostic tests. Could even include appts for post-discharge telephone calls.	0 = facility does not perform this process 1 = this process is performed inconsistently 2 = this process is performed consistently but only for certain disease specific patient groups 3 = this process is done for all patients who meet criteria for receiving this process but is not disease dependent.
Direct communication with PCP or other PACT team members	Direct communication could be through “warm” hand-offs such as phone calls, secure emails, SKYPE/LYNC instant messaging or “cold” hand-offs such as notes or portions of notes designed to proactively address exactly what the PCP needs to address in follow-up	0 = discharge summary only 1 = deliberate hand offs done inconsistently 2 = deliberate hand offs done for certain patient populations 3 = deliberate hand offs done consistently across patients
Need for rehabilitation services routinely assessed during discharge planning	Inpatient physicians, case managers, nurses or pharmacists assess pts. for their potential need for rehabilitation services (PT/OT at home, PT/OT outpatient, inpatient rehabilitation or SNF). This could be done by individuals or in IDTs or medical team rounds.	0 = did not observe in IDT or mention in interviews, 1 = mentioned in interviews but not observed in IDTs 2 = mentioned and observed infrequently in IDTs and/or PTs inconsistently present 3 = PT present or calls in to IDTs and gives input
Assessment for advanced care planning (palliative / hospice)	Patients assessed for quality of life/goals of care and need for palliative care and/or hospice. Applies to patients with severe chronic illnesses such as CHF, COPD, cirrhosis, metastatic cancer, or just complex disease burden.	0 = facility does not perform this process 1 = this process is performed inconsistently 2 = this process is performed consistently but only for certain disease specific patient groups or palliative care in IDTs 3 = this process is done for all patients who meet criteria for receiving this process but is not disease dependent.
Enlisting social and community supports (home health services, Meals-on-Wheels, day care services, housing, etc.) for post-discharge care	Assessing patients’ needs at home as they recover from hospitalization and then referring to community services available to fill those gaps. Most often performed by social work. What is available varies by community.	0 = did not observe in IDT or mention in interviews, 1 = mentioned in interviews but not observed in IDTs 2 = mentioned and observed in IDTs but mostly home health and transportation but not other community services 3 = mentioned and observed both home health and other community services
Post-discharge patient hotline available?	Providing number to patient to call 24 hours a day with questions or concerns post-discharge. Usually manned by nurses.	0 = no hotline 1 = hotline but not staffed by nurses 2 = hotline staffed by nurses and put in every discharge instruction notes 3 = hotline staffed by nurses with physician back-up
Post-discharge home visit available?	Facility offers transitional care programs that can, when deemed appropriate for high risk patients, provide home visits by a VA provider (NP, PA or MD). This is different from contracting for home health care.	0 = not available at this facility 1 = availability inconsistently or low capacity to do home visits 2 = home visits performed consistently but only for certain high risk patient groups (Ex.: age > 75, 3 or more admissions in last year) 3 = home visits available on referral with less restrictive criteria (larger capacity)
Post-discharge phone call from hospital (who, time frame)	Staff member associated with the discharging specialty calls the patient regarding their status, questions, concerns post-discharge.	0 = facility does not perform this process 1 = this process is performed inconsistently 2 = this process is performed consistently but only for certain patient groups (such as moderate to high risk for admission) 3 = this process is done for all patients who meet criteria for receiving this process but is not disease dependent.
Post-discharge phone call from PACT team	Phone call from PACT team member (usually nurse) regarding their status, questions, concerns post-discharge. VA performance measures include need to have patient’s PACT team or surrogate call NOT just any primary care staff and that it occur within 2 business days after discharge.	0 = facility does not perform this process 1 = < 60% completion rate 2 = 60- < 70% completion 3 = greater than or equal to 70%

The outcome measure of risk-standardized readmission rate (RSRR) for the quarter in which the site visit occurred was derived in the following way. First the risk-standardized readmission rate for the 7 quarters (21 months) surrounding the site visit was obtained from the VA Hospital-Wide 30-day Readmission (HWR) Cube, a product of the Veterans Health Administration Support Service Center (VSSC) [26]. The RSRR are available for each of approximately 130 VA acute-care hospitals, on a quarterly basis since the 1st Quarter of Fiscal Year 2014, under the “Readmission Rate Over Time” tab. Because the values of RSRR at each facility as reported by the VHA were quite variable (one or two percentage points) from one quarter to the next, we used linear regression as a technique to smooth the values at the time of visit. A “best-fit” value of RSRR was calculated for each of the 10 facilities by building a linear regression model for seven quarters of the RSRR (using four quarters prior to the on-site survey, the quarter that included the site visit, and two quarters after the site visit). The predicted value at the quarter of the site visit was calculated from the linear regression model. The RSRR reported by VA is calculated as the (observed readmits / expected readmits) * (the national average raw readmission rate for all VA acute care hospital patients). The expected readmits is based on 40 demographic and medical conditions of the patient, using guidelines from the Agency for Healthcare Research and Quality Clinical Classification Software. The calculation of the RSRR at each hospital was for all short-term acute care discharges including surgical cases but excluding discharged against medical advice, admitted for primary psychiatric diagnoses, rehabilitation, nursing home or treatment for cancer, non-short term acute care stay of ≥365 days, death discharge (death within 1 day of discharge), and planned readmissions. Although we would have preferred to be able to exclude the surgical discharges, it would have required a substantial data collection effort (not automated in the cube) which we estimated would adjust the RSRR up or down by only a few tenths of a percentage point.

Simple linear regression of the Checklist Score as the sole predictor (no additional covariates) of RSRR was performed. In addition, the scores of each individual process were checked for correlation with the RSRR using simple linear regression. Processes with an R^2^ value above 0.3 were used to find the best fit multiple linear regression model (without interaction), based on the adjusted R^2^ statistic which penalizes additional factors which contribute little improvement in the model. For this secondary analysis, no adequate models were found. A scatter plot of the data with the regression equation superimposed was prepared using Excel, and the *p*-value for the linear regression model, the value of R^2^, and diagnostic plots were prepared within SAS version 9.4 [27]. All analyses were performed using either SAS version 9.4 [27] or the R statistical package, version 3.4.4 [28], including the “leaps” package [29].

## Results

The degree of implementation of the 20 transitional care processes to prevent readmission varied across our 10 case study facilities. Table 2 shows scoring of process performance at each facility.

**Table 2:** Transitional Care Process Site Scores

Site	1	2	3	4	5	6	7	8	9	10	AV (Range)	# Facilities scored 3	# Facilities scored 0
**Process Name**	**Site Score**												
Pre-discharge patient education	3	3	3	3	3	3	3	3	3	3	3	10	0
Medication reconciliation prior to discharge	3	3	3	3	3	3	3	3	3	3	3	10	0
Implementation of discharge planning rounds	3	1	3	3	3	3	3	3	3	3	2.8 (1–3)	9	0
Assignment of medication reconciliation to pharmacist	0	3	3	3	3	3	3	3	2	3	2.6 (0–3)	8	1
Enlisting social and community supports (home health services, Meals-on-Wheels, day care services, housing, etc.) for post-discharge care	2	1	3	3	3	3	3	3	3	3	2.7 (1–3)	8	0
Printed follow-up instructions which might include medication reconciliation, follow-up appointments, self-care tasks or action plan for management of symptoms	2	3	2	2	3	3	2	2	3	2	2.4 (2–3)	5	0
Post-discharge phone call from PACT team	1	3	3	3	1	3	3	1	2	2	2.2 (1–3)	5	0
Implementation of a discharge checklist	0	1	1	3	3	3	1	3	3	0	1.8 (0–3)	5	2
Utilization of discharge/care transitions case manager	2	3	1	3	3	2	2	2	2	3	2.3 (1–3)	4	0
Post discharge follow-up appointments to PCP and for diagnostic testing made prior to discharge	1	1	2	3	3	1	0	1	3	1	1.6 (0–3)	3	1
Direct communication with PCP or other PACT team members	1	3	2	2	2	2	3	3	2	2	2.2 (1–3)	3	0
Need for rehabilitation services routinely assessed during discharge planning	3	1	1	3	3	2	2	1	2	2	2.0 (1–3)	3	0
Increased emphasis on patient education about diagnoses, self-management and medications throughout hospitalization	0	1	0	0	1	0	2	3	1	1	.7 (0–3)	1	4
Assessment for advance care planning (palliative / hospice)	1	1	2	1	1	1	2	1	3	1	1.4 (1–3)	1	0
Post-discharge patient hotline available?	0	2	2	2	3	2	2	2	2	2	1.9 (0–3)	1	1
Post-discharge home visit available?	0	2	2	0	0	0	0	3	1	2	1.0 (0–3)	1	5
Post-discharge phone call from hospital (who, time frame)	0	0	0	2	3	0	1	0	0	2	0.8 (0–3)	1	6
Communication of medical plans in front of patients during physician team rounds	0	2	2	0	2	2	2	2	2	2	1.6 (0–2)	0	2
Use of teach-back method with patients	2	2	1	2	2	1	2	1	2	2	1.7 (1–2)	0	0
Assessment of readmission risk	0	0	1	1	2	0	0	0	0	0	0.4 (0–2)	0	7
**Summary Score**	**24**	**36**	**37**	**42**	**47**	**37**	**39**	**40**	**42**	**39**	**38.3 (24–47)**		
**Best Fit Predicted RSRR**	**16.1**	**15.2**	**14.7**	**12.8**	**13.0**	**14.1**	**11.9**	**13.1**	**11.8**	**12.9**			

Total scores ranged from 24 to 47 with a mean of 38. No site performed all 20 processes for all applicable patients. Table 2 displays the processes in order of decreasing number of facilities that performed the procedures for all patients (score of 3). Only two processes (pre-discharge patient education, medication reconciliation prior to discharge) were observed occurring for all patients at all facilities. Three processes were performed for all patients in the large majority of facilities (8–9/10): implementation of discharge planning rounds, assignment of medication reconciliation to pharmacist, and enlisting social and community supports (home health services, Meals-on-Wheels, day care services, housing, etc.) for post-discharge care. Three processes were performed for all patients in half of the facilities: printed follow-up instructions (which might include medication reconciliation, follow-up appointments, self-care tasks or action plan for management of symptoms); post-discharge phone call from PACT team; and, implementation of a discharge checklist. Two of these were performed in all facilities at least some of the time (printed follow-up instructions; post-discharge phone call from PACT team) but implementation of a discharge checklist was not done at all in two facilities. Four processes were done for all patients in just 3–4/10 facilities: utilization of discharge/care transitions case manager; post discharge follow-up appointments to PCP and for diagnostic testing made prior to discharge; direct communication with PCP or other PACT team members; and need for rehabilitation services routinely assessed during discharge planning. The remaining eight processes in the table were performed for all patients in only one facility (*n* = 5) or no facilities (*n* = 3).

**Fig. 1:**
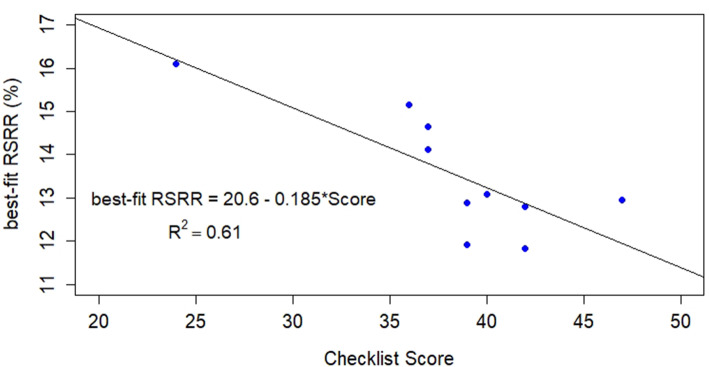
Checklist Score by Risk Adjusted Readmission Rate

Total care transition process scores were linearly correlated with best-fit predicted RSRR (R^2^ = 0.61, *p*-value = 0.007) (see Fig. 1). According to the model, for each one-point increase in transition process score, the RSRR was reduced by 0.185 percentage points. For example, a 10 point difference in total score would account for a 1.85% difference in readmission rate. The linear model showed no evidence of heteroscedasticity using White’s test (*p*-value = 0.63). The studentized residuals were well within the range of − 2 to + 2, and the Cook’s D statistic for influential points only indicated one point of possible concern, facility 5 (with the highest checklist score) with a Cook’s D of 0.45 compared to the conventional cutoff of 0.4. The data point for facility 1 on RSRR did not exceed the Cook’s D cutoff.

No individual processes were reliably correlated with best fit RSRR, e.g. the Cook’s D was out of range for the only two that had significant correlation. Stepwise regression techniques choosing the four processes with the lowest *p* value resulted in an overfitted model that likely would fit only these ten facilities and not be generalizable.

## Discussion

While the extent to which readmissions are preventable is still widely debated [8], the medical and social complexities of patients with highest risk for readmission are now recognized [10, 30] and hospitals have generally focused attention on improving care transitions as a strategy to reduce preventable readmissions. Despite this, US healthcare systems still struggle with the lack of coordination between inpatient and outpatient care [31], primary care and specialty care, home health and rehabilitation services, and social support beyond traditional medical care [32]. While risk-adjusted readmission rates have fallen over the last decade, the disproportionate decrease in adjusted rates versus crude readmission rates may be more related to risk adjustments and use of observation units than to improved transitional care [7, 20, 22, 23, 30].

Despite published suggestions for evidence-based components for transitional care services (pre-discharge, at discharge and post-discharge or within the domains of the transitional care framework) [9, 14–17]_,_ and widespread promotion of models designed to reduce readmission rates [12, 31], the actual number and content of the services delivered may deviate greatly from recommendations. Our data suggest a continuing high degree of variability between facilities around the number of transitional care services provided and to whom they are provided even in the largest single health care system in the US. While some of this variability may reflect potentially appropriate adaptation of processes to local context, this variability may influence processes’ effectiveness. Additionally, the data presented here do not address quality, timing and fidelity of these services which are likely to contribute to further variability.

Our results suggest that consistently providing a greater number of the recommended evidence-based transitional care processes may reduce readmissions. These results confirm previous similar observations by Leppin [14], Burke [18], and Bradley [19]. Burke found that the more domains of the ideal transitions in care framework were addressed in an intervention, the more likely it was to significantly reduce readmissions [18]_._ However, most published interventions on average included only 3.5 of the 10 domains. Bradley [19] found that for care transitions interventions for CHF and AMI, the higher number of strategies employed (out of 10 assessed), the larger the impact on readmissions.

While some processes have been adopted widely and targeted at all patients being discharged, others processes have not. Eight of the 20 processes we examined were either not observed at all or were observed only for a select few patients. All of these processes have convincing evidence for their impact on readmissions. So why have they not been adopted? Some may require significant change in how we think about clinicians’ primary responsibilities and competing priorities. For example, use of teach-back was frequently endorsed as an ideal taught in nursing school but rarely used amid the reality of patient load and time constraints; nurses interviewed perceived time limitations—e.g. it took too much time to wait for the patient to repeat back their knowledge of the instructions. Similarly, ensuring patient education throughout the hospitalization was also acknowledged as a goal, but often fell short due to local rules requiring physician orders for the education, concerns that ill patients were not ready for education until late in the hospitalization, or reliance on future technology (such as teaching videos on closed circuit hospital TV network) that would deliver the education rather than being delivered one-on-one by staff. The low rates of assessment for palliative or hospice care suggests that there may be a gap in clinicians’ recognition that many of the diseases for which patients are repeatedly readmitted are end-stage in nature and/or that palliative consultation might now be appropriate. Burke [18] found that advanced care planning was not included in any of the 66 care transitions intervention studies they reviewed, suggesting the designers of the studies failed to consider advanced care planning as an intervention for reducing readmissions either. O’Connor [33] and Ranganathan [34] found an association between palliative care consultations, especially those involving goals of care discussions and lower readmission rates. A lack of continuity of care between inpatient and outpatient settings, and/or the lack of comfort many providers feel with end-of-life discussions, may further inhibit palliative assessments. One particularly surprising finding was how infrequently formal assessment of risk for readmission was performed. The utility of these instruments for informing transition of care decisions weighed against the time to run them for patients as well as both the interpretability and actionability of the results were cited as a reason for abandoning their use in at least two sites. Finally, barriers to real-time, interprofessional communication may prevent adoption of processes like communication with outpatient clinicians.

We note several limitations to our study. This is a 10-site observational study in a single health care system. Our results may not apply broadly to all health care systems. Our data focuses on transitional care practices for all patients admitted to medical acute units, not just high-risk patients. Although our observations were limited to a subset of inpatients hospitalized during site visits, triangulation with interview data and member checks helped to validate our scoring of processes. Many of these transitional care processes, which we rated using a simplified categorical system (0–3), are complex. Our rating system was only on a single dimension (were the processes performed and, if so, were they targeted to some or all of the patients). We recognize that this system did not explicitly rate the processes on intensity, quality or other unrecognized dimensions. We suspect findings may be somewhat conservative due to minimizing the impact of these other qualities on the outcomes. Higher quality or higher intensity services could reduce readmissions more. Our choice of processes was derived from a review of the literature prior to data collection, selecting those processes which had been shown to have a statistically significant effect on readmissions. We acknowledge that we may not have captured all effective processes, especially those shown to be effective after the start of data collection. These could include having a dedicated discharge coach, linkage to community health workers, comprehensiveness and timeliness of discharge summary, post-discharge monitoring plan, explicit involvement of caregivers in education, and specific actions to take in high-risk patients. Given our small sample size, some of our findings could be driven by outliers in our sample. We tested for this in our model. Site 5 was the only one that tested slightly over the limits of the Cook’s D statistic (.45 vs. .40 as the limit) for its checklist score. No facilities tested as outliers for RSRR. Additionally, our analysis is correlative and does not prove causality. Finally, factors not measured in our study such as organizational culture could also influence readmission rates and covary with processes which we did observe, such as communication of medical plans in front of patients. Brewster [35] in a qualitative study identified 4 organizational practices associated with hospitals with lower readmission rates including: improving collaboration across disciplinary boundaries within the hospital, building relationships to share hospital expertise with postacute providers, enthusiasm for trial and error learning, and fostering a shared sense that readmissions were bad for patients. Future work should explore organizational culture as a factor in readmissions.

## Conclusions

Performing all recommended care transition processes consistently and for all patients for which they are applicable may have potential to further reduce early readmissions. Given the variability in implementation and barriers noted by clinicians to consistently perform processes, further reduction of readmissions will likely require new strategies to facilitate implementation of these evidence-based processes, should include consideration of how to better incorporate activities into workflow and reduce siloing, and may benefit from more consistent use of all of some of the more underutilized processes including patient inclusion in discharge planning and increased utilization of community supports. Although all facilities had inpatient social workers and/or dedicated case managers working on transitions, many had none or limited true bridging personnel (following the patient from inpatient to home and even providing home visits). More investment in these roles may also be needed.

## Data Availability

Sources of VA operational data are cited in references [21, 22]. Investigators must obtain VA research privileges to access the data. Qualitative datasets from the study can be obtained by request from the corresponding author.

## Abbreviations

HRPP: Hospital Readmission Reduction Program
RD: Registered Dietician
RN: registered nurse
RSRR: risk standardized readmission rate
VA: US Department of Veterans Affairs
VSSC: Veterans Health Administration Support Service Center

## References

[1] Ashton CM, Kuykendall DH, Johnson ML, Wray NP, Wu L (1995). The association between the quality of inpatient care and early readmission. Ann Intern Med.

[2] Weissman J, Ayanian J, Chasan-Taber S, Sherwood MJ, Roth C, Epstein AM (1999). Hospital Readmissions and Quality of Care. Med Care.

[3] Centers for Medicare & Medicaid Services. https://www.cms.gov/Medicare/Quality-Initiatives-Patient-Assessment-Instruments/HospitalQualityInits/VA-Data Accessed 5-7-2020.

[4] Centers for Medicare & Medicaid Services. https://www.cms.gov/Medicare/Quality-Initiatives-Patient-Assessment-Instruments/HospitalQualityInits/OutcomeMeasures Accessed 5-7-2020.

[5] McIlvennan CK, Eapen ZJ, Allen LA (2015). Hospital readmissions reduction program. Circulation.

[6] Winblad U, Mor V, McHugh JP, Rahman M. ACO-affiliated hospitals reduced rehospitalizations from skilled nursing facilities faster than other hospitals. Health Affairs . 2017;36:67–73. 10.1377/hlthaff.2016.0759 PMC5553196 28069848

[7] Medicare Payment Advisory Commission. Report to the Congress: promoting greater efficiency in Medicare. http://www.medpac.gov/docs/default-source/reports/Jun07_EntireReport.pdf?sfvrsn=0 . .

[8] Van Walraven C, Bennett C, Jennings A, Austin PC, Forster AJ (2011). Proportion of hospital readmission deemed avoidable: a systematic review. CMAJ..

[9] Burke RE, Kripalani S, Vasilevskis EE, Schnipper JL (2013). Moving beyond readmission penalties: creating an ideal process to improve transitional care. J Hosp Med.

[10] Naylor MD, Aiken LH, Kurtzman ET, Olds DM, Hirschman KM (2011). The importance of transitional care in achieving health reform. Health Aff.

[11] Naylor MD, Brooten D, Campbell R (1999). Comprehensive discharge planning and home follow-up of hospitalized elders. JAMA..

[12] Jack BW, Veerappa KC, Anthony D (2009). A reengineered hospital discharge program to decrease rehospitalization: a randomized trial. Ann Intern Med.

[13] Coleman EA, Parry C, Chalmers S, Min S-J (2006). The care transitions intervention. Arch Int Med.

[14] Leppin AL, Gionfriddo MR, Kessler M (2014). Preventing 30-day hospital readmissions: a systematic review and meta-analysis of randomized trials. JAMA Int Med.

[15] Boutwell A, Hwu S (2009). Effective interventions to reduce rehospitalizations: a survey of the published evidence.

[16] Hansen LO, Young RS, Hinami K, Leung, Williams MV. Interventions to reduce 30-day rehospitalization: a systematic review. Ann Intern Med2011; 155:520–528. 10.7326/0003-4819-155-8-201110180-00008 22007045

[17] Kansagara D, Chiovaro JC, Kagen D (2016). So many options, where do we start? An overview of the care transitions literature. J Hosp Med.

[18] Burke RE, Guo R, Prochazka AV, Misky GJ (2014). Identifying keys to success in reducing readmissions using the ideal transitions in care framework. BMC Health Serv Res.

[19] Bradley EH, Curry L, Horwitz LI, Sipsma H, Wang Y, Walsh MN, Goldmann D, White N, Peña IL, Krumholz LM. Hospital strategies associated with 30-day readmission rates for patients with heart failure. Circ Cardiovasc Qual Outcomes. 2013;6:444–50. 10.1161/CIRCOUTCOMES.111.000101 PMC3802532 23861483

[20] Kaboli PJ, Go JT, Hockenberry J, Glasgow JM, Johnson SR (2012). Associations between reduced hospital length of stay and 30-day readmission rate and mortality: 14-year experience in 129 veterans affairs hospitals. Ann Intern Med.

[21] Boccuti C, Casillas G. Issue brief: fewer hospital U-turns: the Medicare hospital readmission reduction program. The Henry J. Kaiser Family Foundation, 2017.

[22] Wright B, O’Shea AM, Ayyagari P, Ugwi PG, Kaboli P, Sarazin MV. Observation rates at Veterans’ hospitals more than doubled during 2005-2013, similar to Medicare trends. Health Aff. 2015;34:1730–7. 10.1377/hlthaff.2014.1474 26438750

[23] Noel-Miller C, Lind K. Is observation status substituting for hospital readmission? Health Affairs Blog. 2015;28 10.1377/HBLOG20151028.051459.

[24] Penney LS, Leykum LK, Noël P (2018). Protocol for a mixed methods study of hospital readmissions: sensemaking in veterans health administration healthcare system in the USA. BMJ Open.

[25] VHA Support Service Center, Patient Aligned Care Teams Almanac, https://reports.vssc.med.va.gov/ReportServer/Pages/ReportViewer.aspx?%2fPC%2fAlmanac%2fMainMenu&rs%3aCommand=Render Accessed May 7, 2020.

[26] VA Hospital-Wide 30-day Readmission (HWR) Cube, a product of the Veterans Health Administration Support Service Center (VSSC) VSSC URL: (URL: https://bioffice.pa.cdw.va.gov/default.aspx?bookid=99284cd4-f909-4eee-aa8e-49065f12afeb|ispasFalse|report04a80ff9-b3fd-4223-a583-47d3859101d1|ws1|wsb0|isDisabledAnalyticsFalse|isDashboardPanelOnTrue). (Note: the Cube can also be accessed by going to the Quality of Care page on the VSSC website (URL: https://vssc.med.va.gov/VSSCMainApp/products.aspx?PgmArea=82 ) and then clicking on Product Name: Hospital-Wide 30-day Readmission Cube.)

[27] SAS/STAT [computer program]. Version 9.4. Cary, NC: SAS Institute Inc; 2014.

[28] R Core Team (2018). R: a language and environment for statistical computing. R Foundation for statistical computing, Vienna, Austria. URL https://www.R-project.org/.

[29] Thomas Lumley based on Fortran code by Alan Miller (2020). leaps: Regression Subset Selection. R package version 3.1. https://CRAN.R-project.org/package=leaps

[30] Ody C, Msall L, Dafney LS, Grabowski DC, Cutler DM (2019). Decreases in readmissions credited to Medicare’s program to reduce hospital readmissions have been overstated. Health Aff.

[31] Society of Hospital Medicine. https://www.hospitalmedicine.org/globalassets/clinical-topics/clinical-pdf/8ps_riskassess-1.pdf Accessed May 7, 2020.

[32] Jones CD, Vu MB, O’Donnell CM (2014). A failure to communicate: a qualitative exploration of care coordination between hospitalists and primary care providers around patient hospitalizations. J Gen Intern Med.

[33] O’Connor NR, Moyer ME, Behta M, Casarett DJ (2015). The impact of inpatient palliative care consultations on 30-day hospital readmissions. J Palliat Med.

[34] Ranganathan A, Doughterty M, Waite D, Casarett D (2013). Can palliative home care reduce 30-day readmissions? Results of a propensity score matched study. J Palliat Med.

[35] Brewster AL, Cherlin EJ, Ndumele CD, Collins D, Burgess JF, Charns MP, Bradley EH, Curry LA (2016). What works in readmissions reduction: how hospitals improve performance. Med Care.

